# Uterine sarcoma: almost more tumor than patient

**DOI:** 10.1007/s00404-023-07102-9

**Published:** 2023-06-15

**Authors:** Laura Burney Ellis, Benjamin Jones, Itai Yagel, Abigail Walker-Jacobs, Apostolia Galani, Lorraine Hamlyn, Maria Kyrgiou

**Affiliations:** 1 Imperial College London, London, UK; 2 Imperial College Healthcare Trust, London, UK

A nulliparous woman declined a hysterectomy for her 30 cm fibroid uterus in 2018, recommended for radiological suspicion of sarcomatous change, and instead attempted alternative medical remedies. She re-presented 5 years later; computerized tomography imaging identified a massive leiomyoma measuring 42 × 38 × 50 cm, with a 30 cm fluid-filled pocket, and enduring concern of possible sarcomatous change.

She underwent debulking surgery. Intra-operatively, the mass was arising from a stalk at the uterine fundus. It was adherent to the anterior abdominal wall, pelvic side walls, small bowel, large bowel, uterine fundus, and the left ovary, with multiple large feeding vessels. A 10 cm segment of small bowel was excised, and a side-to-side anastomosis created. A total abdominal hysterectomy, bilateral salpingo-oophorectomy, and omentectomy were then completed. The estimated blood loss was 3.5 L; requiring transfusion of 10 units of blood and 4 units of fresh frozen plasma. 8 L of fluid drained from the mass; after removal, total estimated weight of the mass was 45 kg. She was discharged 11 days later, weighing 51 kg, after being admitted at 96 kg. Histology confirmed high-grade leiomyosarcoma (stage II, grade 3).

Leiomyosarcomas of the uterus are rare; affecting around 6 per million people per year in the United States [1]. Staging is according to the Federation of Gynecology and Obstetrics staging of uterine sarcomas [2]. They are aggressive malignancies with a poor prognosis; 5-year survival for those diagnosed at an early stage is estimated to be between 25 and 76% [3]. Surgical treatment is recommended, and adjuvant therapy remains controversial with no proven survival benefit [2]. Recurrence rates are estimated around 45–75% [4].

**Fig. 1:**
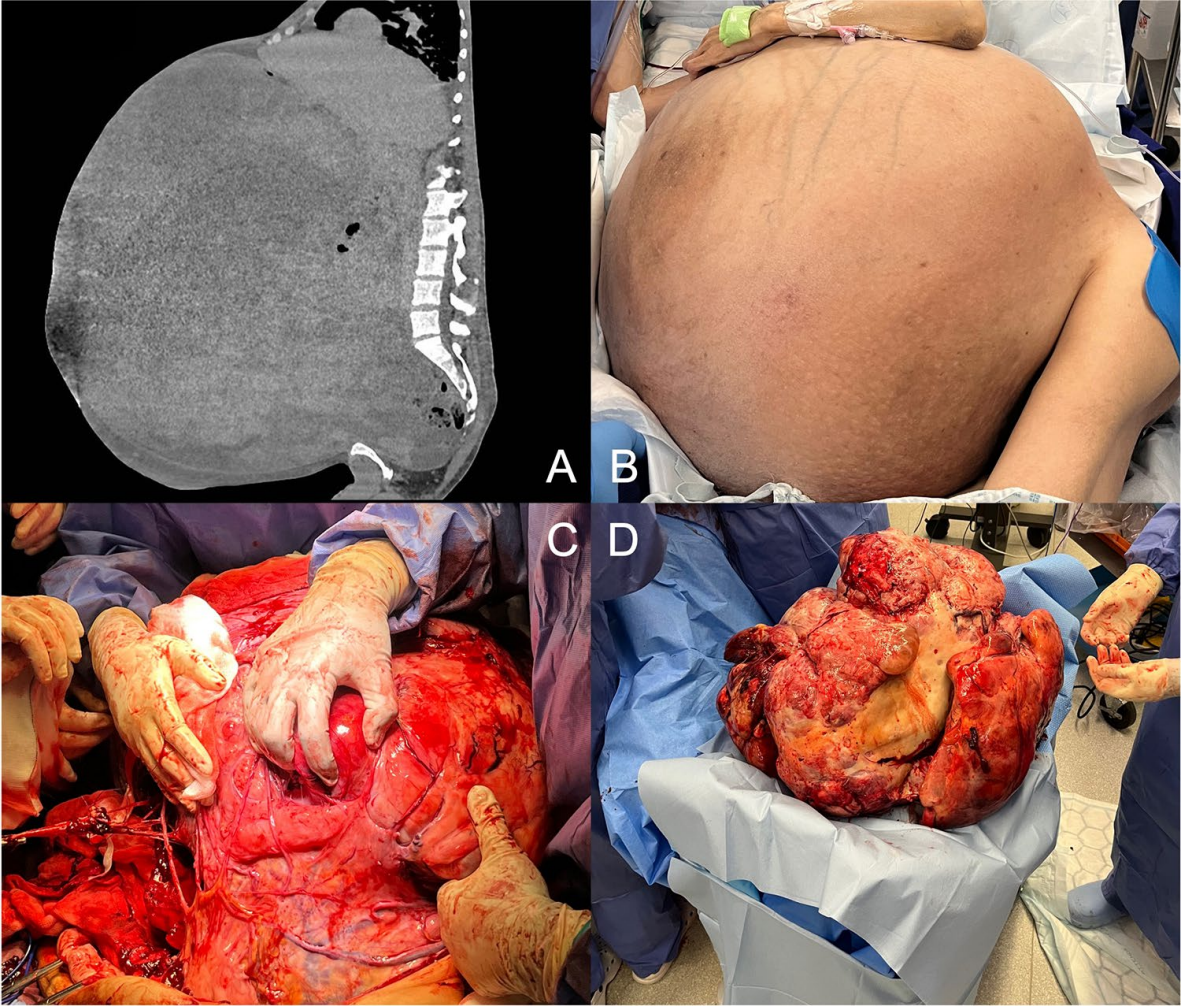
**A** Cross-sectional image displaying abdominal mass 50 cm in diameter. **B** Patient positioning in a right lateral position in the operating theatre. **C** Intra-operative photography displaying the mass and adhesions to surrounding structures including transverse colon. **D** Abdominal mass, once removed, and drained of 8 L of fluid

There have been large leiomyosarcomas reported in the literature; the largest being 57 kg [5], measuring 40 cm, found in a patient with a BMI of 41. At 50 cm, this is the largest documented leiomyosarcoma, remarkably making up 47% of the patient’s pre-operative weight Fig. 1.
